# Molecular Regulatory Mechanism and Toxicology of Neurodegenerative Processes in MPTP/Probenecid-Induced Progressive Parkinson’s Disease Mice Model Revealed by Transcriptome

**DOI:** 10.1007/s12035-020-02128-5

**Published:** 2020-09-30

**Authors:** Weiwei Yang, Wenwen Hao, Zhuo Meng, Shiyan Ding, Xiaodi Li, Tao Zhang, Weixiao Huang, Lian Xu, Yu Zhang, Jian Yang, Xiaosong Gu

**Affiliations:** 1 Key Laboratory of Acupuncture and Medicine Research of Ministry of Education, School of Medicine & Holistic Integrative Medicine, Nanjing University of Chinese Medicine, Nanjing, China; 2 Jiangsu Clinical Medicine Center of Tissue Engineering and Nerve Injury Repair, Co-innovation Center of Neuroregeneration, Nantong University, Nantong, China

**Keywords:** Parkinson’s disease, Neurodegeneration, Molecular toxicology, RNA sequencing, MPTP/p

## Abstract

Parkinson’s disease (PD) is a neurodegenerative disease caused by a variety of unclear complex pathogenic factors. The 1-methyl-4-phenyl-1, 2, 3, 6-tetrahydropyridine/probenecid (MPTP/p)-induced progressive PD mice is a well-recognized classic model for studying PD, but the molecular toxicology of this model is still unclear. Here, for the first time, we report gradual neurodegenerative processes in MPTP/p-induced progressive PD mice model using RNA-seq. Transcriptional responses are orchestrated to regulate the expression of many genes in substantia nigra, such as *Ntf3*, *Pitx3*, *Th*, and *Drd2*, leading to the degeneration of dopaminergic neurons at last. We proposed that the established model could be divided into three phases based on their molecular toxicological features: “the stress response phase” which maintained the microenvironment homeostasis, “the pre-neurodegenerative phase” which demonstrated observed MPTP/p cytotoxicity and gradual degeneration of dopaminergic neurons, and “the neurodegenerative phase” which reflected distinct damage and dopaminergic neuron apoptotic process. Glia cells exhibited a certain protective effect on dopaminergic neurons in 3rd and 6th MPTP/p-induced cytotoxicity. But in 10th MPTP/p injection, glia cells play a promoting role in PD and tissue damages caused by oxidative stress. This study also indicated that the substantia nigra of PD mice showed unique patterns of changes at each stage. Moreover, neurotrophic signaling pathway, ECM-receptor interaction, oxidative phosphorylation, apoptosis and necroptosis were enriched at 3rd and 6th MPTP/p injection, which might be associated with the PD progress. This study provided an extensive data set of molecular toxicology for elucidating of PD progression and offered comprehensive theoretical knowledge for the development of new therapy.

**Electronic supplementary material:**

The online version of this article (10.1007/s12035-020-02128-5) contains supplementary material, which is available to authorized users.

## Introduction

Parkinson’s disease (PD) is one of the most serious neurodegenerative diseases second to Alzheimer’s disease. Symptoms of the PD have a gradual onset and progression that typical clinical manifestations include dyskinesia, quiescent tremor, muscle stiffness, and postural instability [1], seriously affecting patients’ life quality. At present, more than 1.5% of the global population over 65 years old is suffering from PD [2]. The annual direct medical expenses of patients are estimated to exceed $10,000 [3, 4], which causes a heavy burden on families and society. Brain autopsy results of PD patients showed that dopaminergic neurons in the compact part of substantia nigra decreased and striatum dopamine exhausted, resulting in extrapyramidal motor dysfunction [5]. In addition, large amounts of studies have shown that the brainstem, spinal cord, and related cerebral cortex specifically accumulate large amounts of Lewy bodies aggregated by alpha-synuclein [6, 7].

Currently, the dopamine replacement therapy is one of the most common treatment strategies for PD. Deep brain stimulation, stem cell transplantation, gene therapy, rehabilitation therapy, and other non-drug therapies also could be most used clinically [8–12]. Although these therapies can improve the symptoms on some extent, their role in effectively ceasing the progress of PD is unknown. Therefore, PD remains the focus of neuroscientists for a long time in the future. Nowadays, the pathological mechanism of PD is not clear. Existing studies have shown that PD may be closely related to oxidative stress, glutamate receptor abnormality, ubiquitin-protease dysfunction, inflammation and cytokine activation, neurotrophic factor dysfunction, mitochondrial damage, cytoskeleton abnormality, synaptic dysfunction, and apoptotic pathway activation [13–17]. In addition, the pathogenesis of PD may change at different cellular levels, especially at transcriptional level [18]. 1-Methyl-4-phenyl-1, 2, 3, 6-tetrahydropyridine/probenecid (MPTP/p)-induced progressive model in mice is a classical model in the field of PD research, which resembles many of the pathological hallmarks and motor deficits of PD, making it an excellent model for study on pathogenesis [19]. However, the neurodegenerative process of substantial nigra in MPTP/p-induced progressive PD mice is still not clear. Here, we investigated the change in the behavior, neuron morphology, and molecular level of the model after different times of MPTP/p injections with an interval of 3.5 days. Our result showed that the MPTP/p-induced progressive model was successful and reached a stable state after the 10th injection, which was consistent with previous study [20]. Next, we used RNA-seq to investigate the dynamic genetic changes during MPTP/p-induced progressive PD mice modeling.

In this study, we carried out RNA-seq on the tissues of the substantia nigra collected from mice after the 3rd, 6th, and 10th MPTP/p injections and corresponding saline treatment. We explored the potential molecular changes through bioinformatic analysis, including differential expression, expression clustering, functional enrichment, and gene set enrichment. Our results not only showed the studied PD-related gene changes but also revealed the dynamic neurodegenerative processes of MPTP/p-induced progressive PD model in mice. This study provided a valuable resource for understanding the PD progress which would contribute to the development of PD therapy.

## Materials and Methods

**Fig. 1:**
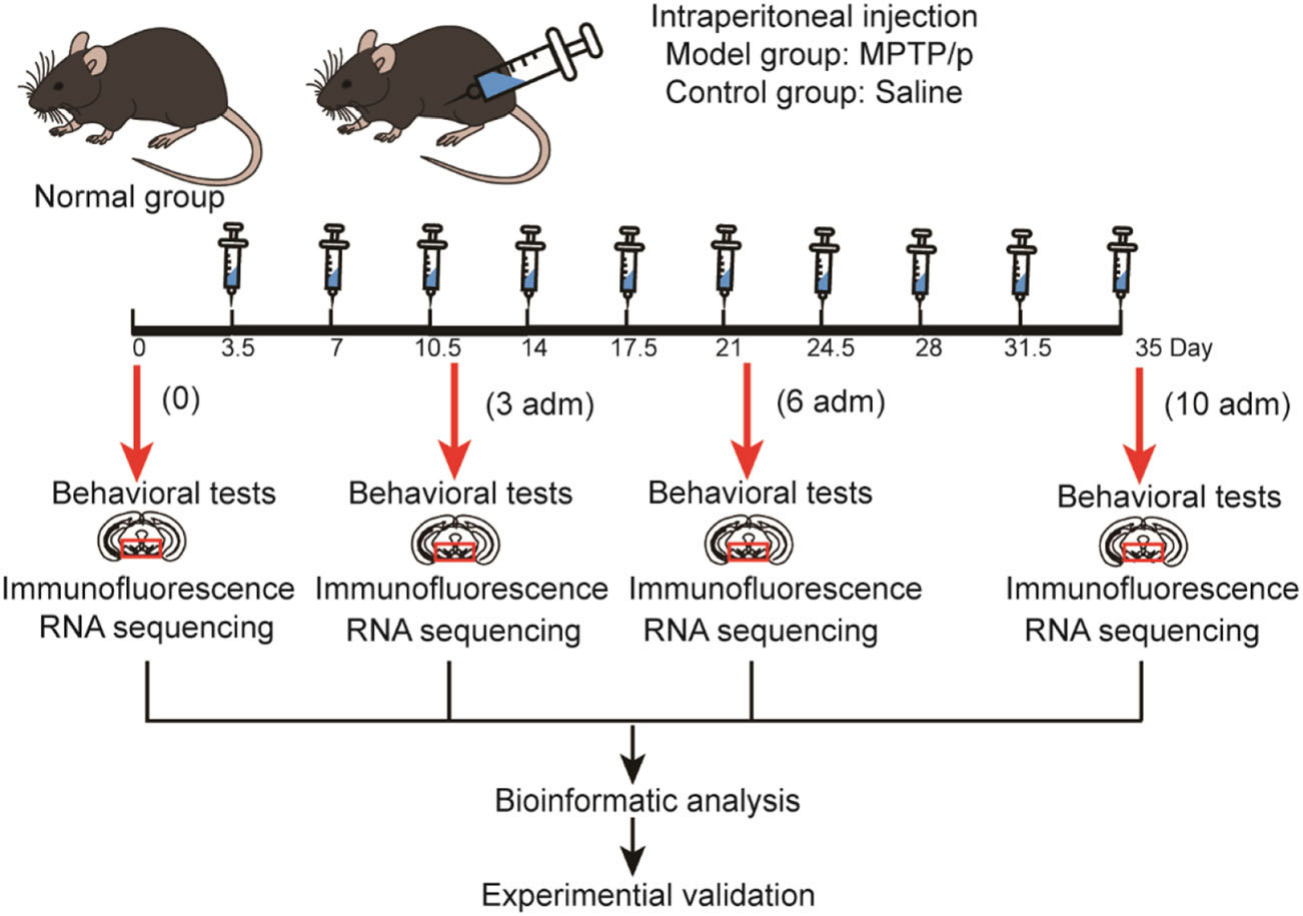
Overview of experiment design in this study

The overview of experimental design in this study was shown in Fig. 1. Progressive PD mice model was established at time-points of 3, 6, and 10 administrations of MPTP/p twice a week for 5 weeks. Behavioral tests and immunofluorescence were used to evaluate behavioral deficits and neurodegeneration. Saline treatment at corresponding time-points was used as control group. Normal group was mice without any treatment. Substantia nigra tissues collected from model, control, and normal groups were used for RNA sequencing respectively. Bioinformatic analysis was used to explore molecular changes during progressive models.

### Animals and MPTP/p Administration

Twelve-week-old male C57BL/6J mice, weighting 25–30 g, were obtained from the Model Animal Research of Nanjing University. Procedures for animal care described herein were in accordance with the Institutional Animal Care and use guidelines of Nanjing University of Chinese Medicine and approved ethically by the Administration Committee of Experimental Animals, Jiangsu Province, China. C57BL/6J mice were randomly assigned to three groups: (i) normal group without any treatment, (ii) model group + (25 mg/kg in saline, Sigma-Aldrich, St. Louis, MO, USA) of MPTP administration and (250 mg/kg in 5% NaHCO_3_, Sigma-Aldrich) of Probenecid, and (iii) control group + (an equal volume of 0.9% sodium chloride) of saline administration. Mice in both model group and control group received injection twice a week for 5 weeks and were handled in accordance with the published guidelines [19, 20]. Mice were kept in an ambient temperature of 22 °C, 12-h light-dark cycle (7:00 am onset), and free access to food and water.

### Behavioral Tests

Olfactory function and motor performance were evaluated at progressive time-points (after 3rd, 6th, and 10th administrations of MPTP/p) in model and control groups, respectively. To avoid the acute pharmacological actions of MPTP/MPP^+^ to neurons, behavioral tests were examined the next day after each time point. Olfactory test was carried out in a clean plastic cage. There were 26 mice in model group and 11 mice in control group, respectively. Mice were food-deprived for 20 h before test. A cheese pellet was buried under the bedding at one of the five selected corners in a cage, and the mouse was positioned in the center of cage at the beginning of test. We measured the time it spent to retrieve and bite the pellet with a maximum duration of 180 s as latency durations. The latency duration for mice to find and bite the cheese in each group was measured and recorded at different time points to evaluate olfactory function. The Beam traversal test was carried on a Plexiglas beam which consisted of four sections (25 cm each, 1 m total length) of different widths, starting at a width of 3.5 cm and gradually narrowing to 1 cm. Mesh grid (1 cm square) of corresponding width was placed over the beam, leaving a 1-cm space between the grid and the beam surface. There were 38 mice in model group and 21 mice in control group, respectively. Mice were trained to traverse the beam from the widest to the narrowest side in 2 consecutive days, and 5 trials per day. On the test day, we recorded the video when mice traversed the grid-surfaced beam for a total of five trials. Videos were recorded and rated in slow motion to count error steps. An error consisted of limbs slipping through the grid during a forward movement that was visible between the grid and the beam surface. The number of error steps of mouse in each group was measured and recorded at different time points to evaluate motor dysfunction. The data in Behavioral tests were expressed as mean ± SD.

### Immunofluorescence

Mice were anesthetized at progressive time points (after 3rd, 6th, and 10th administrations of MPTP/p) and transcardially perfused with 4% paraformaldehyde in 0.1 mol/L PBS (PH7.2). Brain was removed from the skull, soaked in 4% PFA/PBS for 24 h, transferred it into 20% and 30% sucrose solution in 0.1 mol/LPBS for gradient dehydration, and stored at 4 °C. Continuous coronal sections (12 μm thick) of mice brain were prepared in the freezing microtome. For the SNpc, consecutive sections were collected starting at − 2.85 mm anterior from bregma to the posterior ending of the area (75 total sections). SNpc were sectioned into 12 series of 12 μm coronal sections, and every set provided a representative survey of the entire SNpc. Every sixth other section was processed and analyzed for TH immunofluorescence in order to evaluate the loss of dopaminergic neurons. The brain slices were then rinsed in PBS (3 × 10 min) and blocked with 5% bovine serum albumin (Vector Laboratories, Burlingame, CA, USA), 0.5% Triton X-100 in PBS for 1 h at room temperature. Thereafter, the selected slices were incubated with primary anti-TH rabbit polyclonal antibody (1:1000; HPA061003; Sigma-Aldrich, St. Louis, MO, USA) at 4 °C overnight, rinsed in PBS (3 × 10 min), treated with goat anti-rabbit IgG Alexa Fluo 488 (1:1000; 150,077, Abcam, Cambridge, MA, USA) for 2 h at room temperature, rinsed in PBS (3 × 10 min), and incubated with Hoechst33342 (1:2000, Abcam) for 10 min.

### Sample Library Preparation and RNA Sequencing

Total RNA was extracted from the substantia nigra tissues by Trizol (Invitrogen, Carlsbad, CA, USA) according to manual instruction. Subsequently, total RNA was qualified and quantified using a Nano Drop and Agilent 2100 bioanalyzer (Thermo Fisher Scientific, MA, USA). Then treatment of total RNA was carried by mRNA enrichment, and the random N6 primer was used for the obtained RNA reverse transcription. The ligation product was PCR amplified by specific primers. The PCR product was heat-denatured into a single strand, and a single-stranded DNA was cyclized with a bridge primer to obtain a single-stranded circular DNA library. Sequencing was carried on the machine BGISEQ500 platform (BGI-Shenzhen, China).

### Bioinformatics Analysis

Low-quality reads (adapter, Ns ≥5%, *Q*_10_ ≥ 20%) were filtered using Trimmomatic (v0.36) with parameters “ILLUMINACLIP: 2: 30: 10 LEADING: 3 TRAILING: 3 SLIDINGWINDOW: 4: 15 MINLEN: 50” [21]. High-quality reads were mapped to the mouse reference genome retrieved from the NCBI (*Mus musculus*, GRCm38.p5) using Bowtie2 (v2.2.5) [22]. Gene expression quantification was conducted using RSEM (v1.2.8), and gene expression was normalized as fragments per kilo-base per million mapped reads (FPKM) [23]. The differential gene expression analysis of RNA-seq data was performed using DEGseq [24] between model and control groups. Genes with fold-change and adjusted *P* value with $\mid {\log}_2^{\mathrm{fold}-\mathrm{change}}\mid \ge 1\ \mathrm{and}\ P\le 0.001$ between two samples were considered as differential expression. The numbers of differentially expressed genes (DEGs) between model and control groups after progressive time-points (after 3rd, 6th, and 10th administrations of MPTP/p) were visualized using UpSetR package in R [25]. Gene expression clustering was performed to infer expression patterns of DEGs during MPTP/p injection using Mfuzz [26]. Functional enrichment analysis of DEGs identified at the specified time point was based on hypergeometric distribution, and *P* value was corrected with FDR algorithm. The network between KEGG pathway and DEGs was visualized using Cytoscape (v3.7.2) [27]. To further identify genes that were deemed related to PD, we first retrieved PD-related genes from STRING disease query (downloaded in 20190919) using Cytoscape and then performed gene set enrichment analysis with GSEA (v4.0.1) using *t* test as Ranked list metric [28, 29]. Profile of the running ES score and positions of geneset members on the rank ordered list were plotted using the ggplot2 package from R.

### Quantitative Real-Time PCR

To validate the accuracy of RNA-seq, the expression of six genes, including *Th*, *Slc6a3*, *Ntf3*, *Col6a2*, *Fn1*, and *Gfap*, were selected and quantified using RT-qPCR. The reverse-transcribed cDNA was synthesized with the HiScript II Q RT SuperMix for qPCR (+gDNA wiper) (R223-01, Vazyme, Nanjing, China). PCR was performed with ChamQ SYBR qPCR Master Mix (Q311-03, Vazyme) on LightCycler® 480 II Real-time PCR Instrument (Roche, Swiss). For each cDNA, three replicates were performed. The sequences of primer pairs were listed below and synthesized by Generay Biotech (Generay, Shanghai, China):

- *Th*: 5′-GCACACAGTACATCCGTC-3′, 3′-TGGGAGAACTGGGCAAAT-5′,
- *Slc6a3*: 5′-CTGGTGCTGGTCATTGTT-3′, 3′-GCAGGGCTGTGAGGACTA-5′,
- *Ntf3*: 5′-GCAACGGACACAGAGCTACT-3′, 3′-AATGGCTGAGGACTTGTCGG-5′,
- *Col6a2*: 5′-CACTGTGGGAAGGTAGCCAG-3′, 3′-AGGCTGGAGGAAGTAGGAGG-5′,
- *Fn1*: 5′-TGAGCGAGGAGGGAGATGAA-3′, 3′-TAGGTGCCTGGGGTCTACTC-5′,
- *Gfap*: 5′-CACCAAACTGGCTGATGT-3′, 3′-GGTCCTGTGCAAAGTTGT-5′,
- *Gapdh*: 3′-GCAAGGACACTGAGCAAGA-5′, 3′-GGATGGAAATTGTGAGGGAG-5′.

The relative mRNA level was calculated by equation ${2}^{-\Delta  \Delta  {C}_T}$. *Gapdh* was used as an internal control. The data in RT-qPCR were expressed as mean ± SEM. The level of the genes studied in the normal group was set as 1.

## Results

### Behavioral Tests

**Fig. 2:**
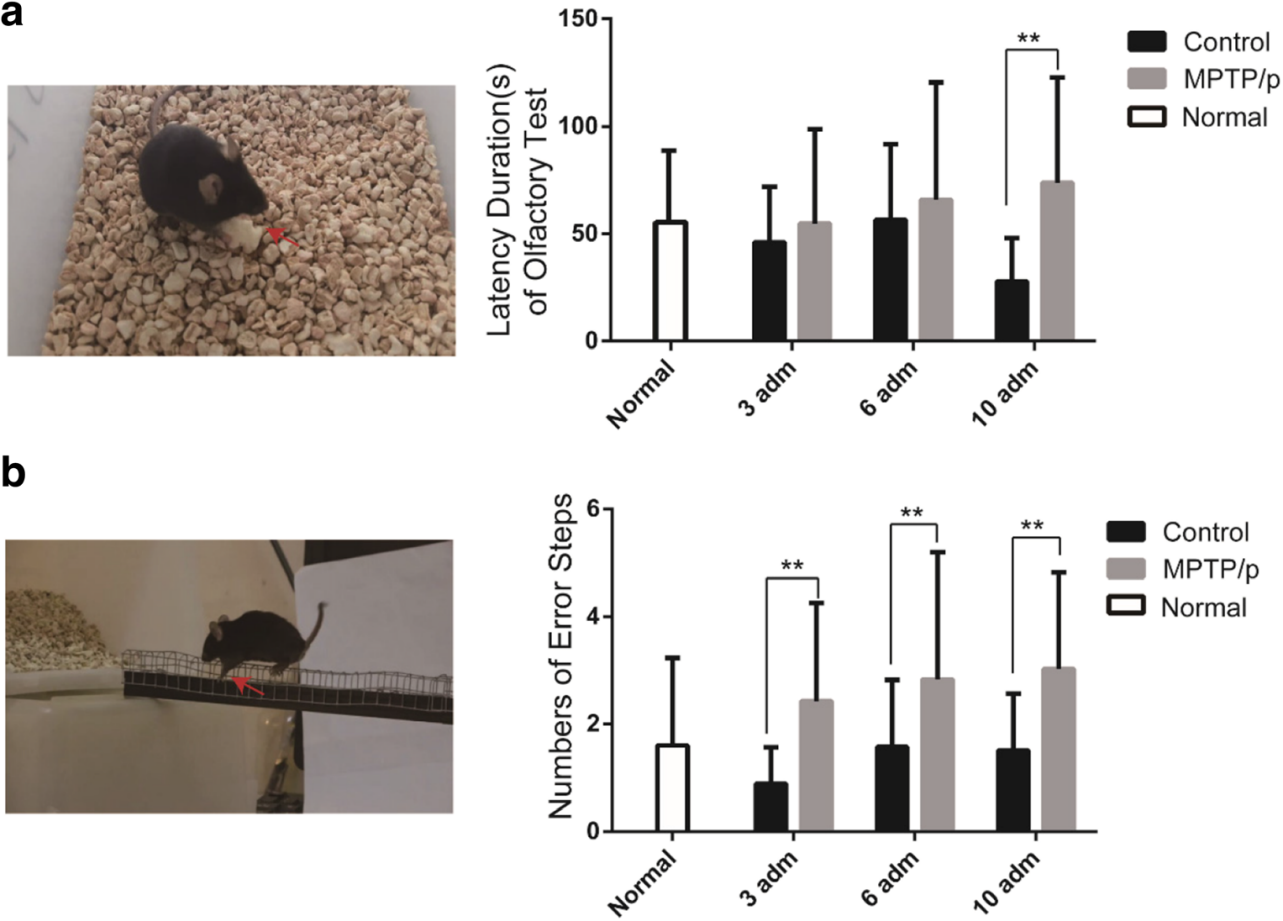
The behavioral tests during the MPTP/p administration. **a** The olfactory test showed significant difference between model group (*n* = 26) and control group (*n* = 11). **b** The beam traversal test pointed out significant differences between model group (*n* = 38) and control group (*n* = 21) at 3rd, 6th, and 10th administration (*P* < 0.01)

Olfactory impairment is considered as an early symptom of PD. The olfactory test showed a significant effect of the treatment on the retrieval-time after the 10th MPTP/p administration compared with the saline-treated mice (*P* < 0.01; Fig. 2a). Outcomes from the beam traversal test showed a significant increase of the number of error steps after the 3rd, 6th, and 10th MPTP/p injections compared with corresponding control groups (*P* < 0.01; Fig. 2b). Thus, behavioral tests indicated the olfactory and motor functions were gradual impairment in MPTP/p-induced progressive PD mouse model, and the behavior was significantly declined after the 10th injection.

### Immunofluorescence

**Fig. 3:**
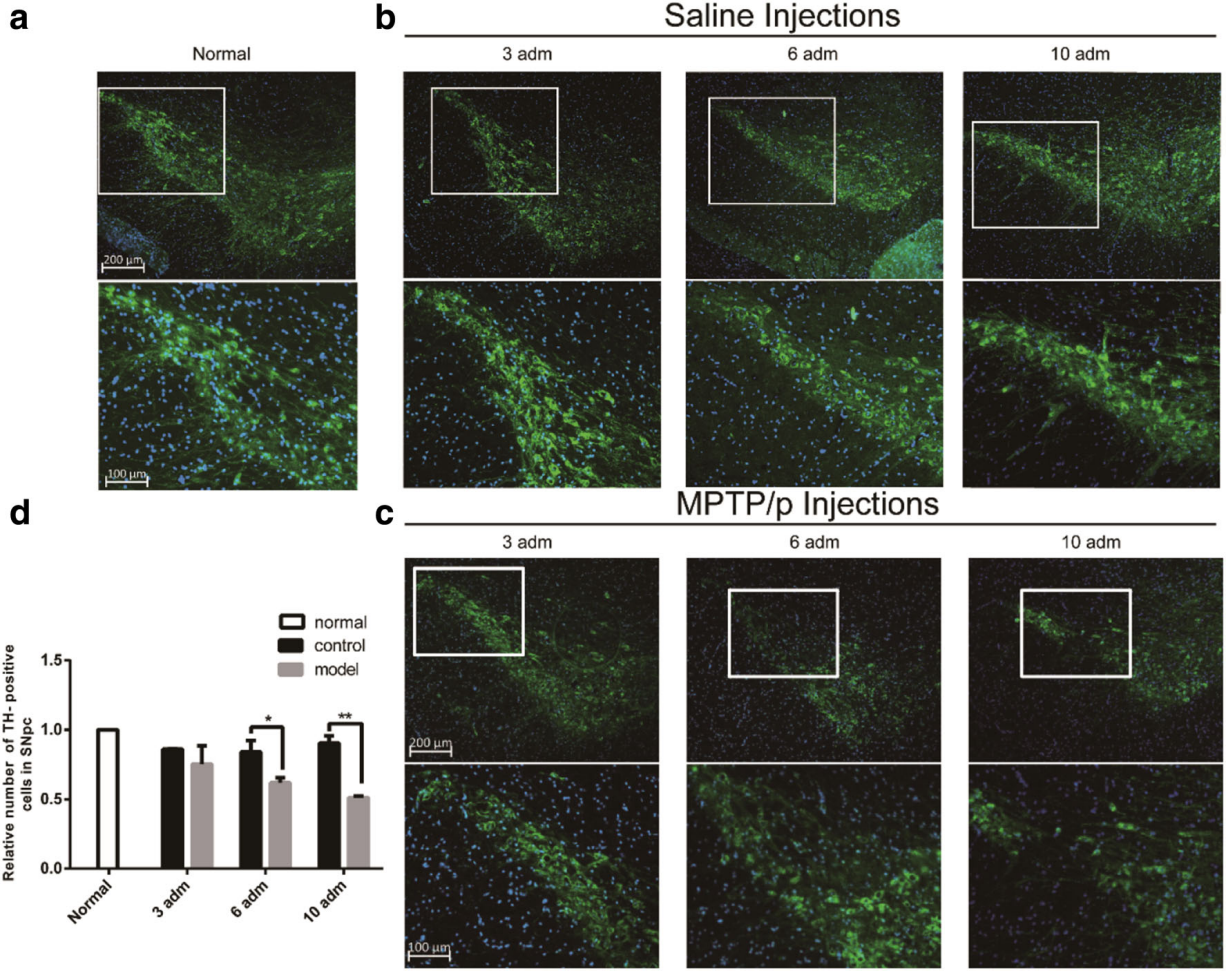
Investigation of TH-positive cells during different times of MPTP/p injection. **a** Immunofluorescence in normal group. **b** Immunofluorescence in control group. **c** Immunofluorescence in model group. **d** Relative number of TH-positive cells in SNpc. Immunofluorescence by anti-TH (green) primary antibody merged by Hoechst 33342 (blue) obtained after 3rd, 6th, and 10th (namely, 3 adm, 6 adm, 10 adm in figures) episodes of injection and normal sample in the substantia nigra frozen section

We counted TH-positive cells in substantia nigra under a 10-fold objective lens. Our result showed that the number of TH-positive cells was not significantly reduced in the saline control group as compared with the normal group (Fig. 3a–b). Conversely, the number of TH-positive cells in MPTP/p-induced PD mice gradually decreased, with the greatest decrease after the 10th injection (*P* < 0.01), along with the increase of MPTP/p administrations (Fig. 3c). Interestingly, there was no obvious decline on the TH-positive cells in substantia nigra after the 3rd MPTP/p injection in the model group compared with the control group (Fig. 3d).

### RNA-seq Analysis

A total of 21 samples were sequenced, yielding an average of 7 gigabytes (Gb) data. The average of genome mapping rate and gene mapping rate was 95.38% and 76.95%, respectively. We detected a total of 20,717 expressed genes for subsequent analysis.

### Global Overview of Differentially Expressed Genes in SNpc After Different MPTP/p Injections

**Fig. 4:**
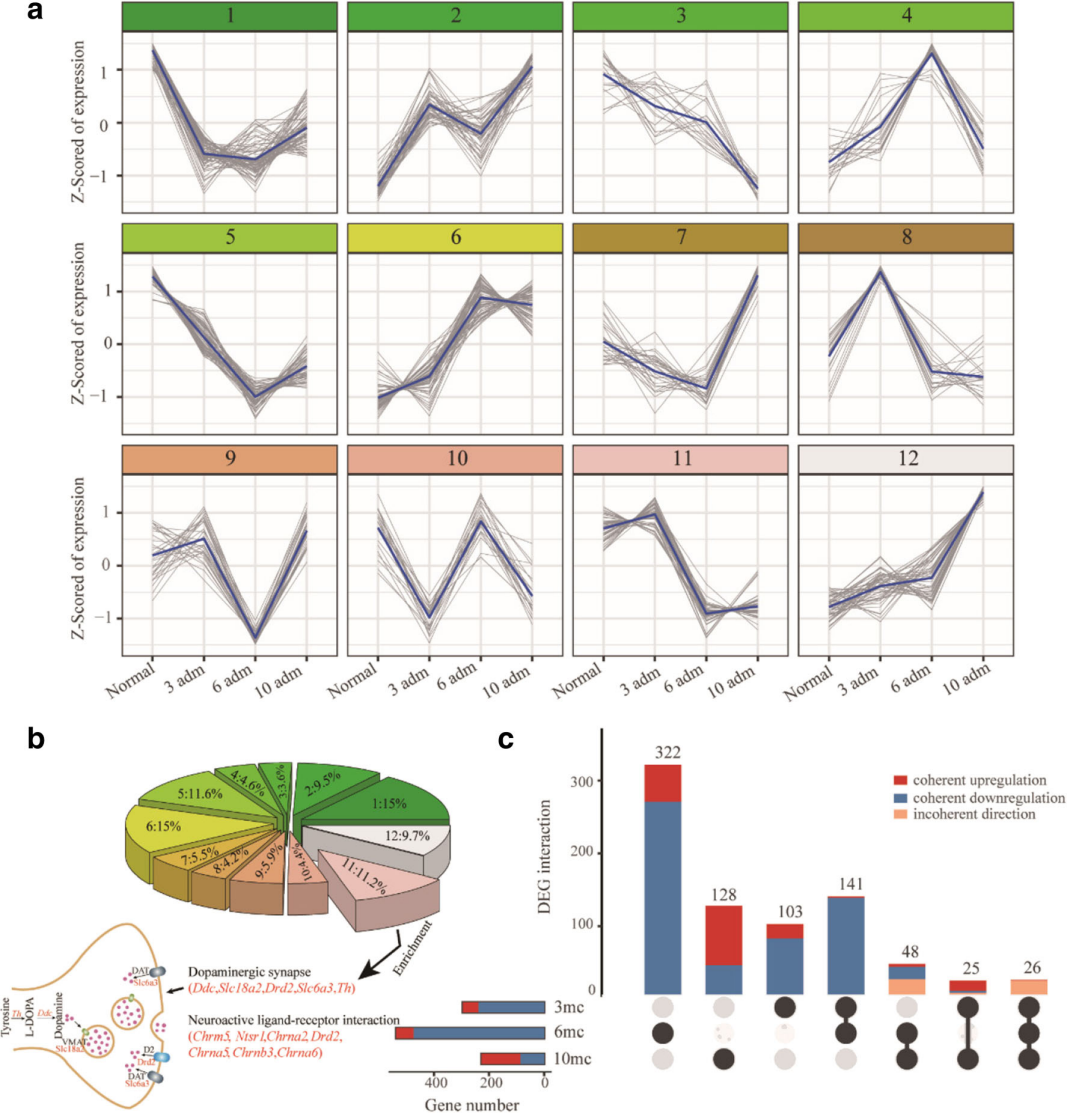
Differentially expressed genes between the MPTP/p injection and saline injection. **a** 12 distinct expression patterns of DEGs at specific time points in model and normal groups. Blue line indicated the average expression value. **b** The ratio of gene number in each cluster from **a** panel and KEGG pathway enrichment of genes in cluster 11. Significant enrichments of KEGG pathways were shown. Genes involved in dopamine synthesis were down-regulated in 6th and 10th injections. **c** The UpSet plot of DEGs between the MPTP/p-induced progressive PD mice model group and the control group after the 3rd, 6th, and 10th injections. “mc” indicated the comparison of model and control groups

DEGs were detected by comparing the model group (MPTP/p treatment) and control group (saline treatment) at time-points 3rd, 6th, and 10th. To overview expression patterns of DEGs in normal and model groups, we employed a soft clustering strategy using Mfuzz, yielding a total of 12 distinct patterns (Fig. 4a and b). Approximate 45.6% of DEGs showed decreased expression at 6th and 10th (clusters 1, 3, 5, 8, and 11; Fig. 4a). Functional enrichment of genes in each cluster showed “dopaminergic synapse,” “neuroactive ligand–receptor interaction” which were significantly enriched from cluster 11 (Bonferroni corrected *P* < 0.05; Fig. 4b). Genes enriched in “dopaminergic synapse” were involved in the synthesis (*Th* and *Ddc*) and transporter (*Slc6a3*, *Slc18a2* and *Drd2*) of dopamine (Fig. 4b). This indicated that dopamine was decreased significantly with the increase of MPTP/p treatment, which was consistent with our result of immunofluorescence. We then analyzed the DEG numbers in each compared group and identified 295, 537, and 227 in the PD mice at the 3rd, 6th, and 10th MPTP/p injections, respectively (Fig. 4c). In addition, we also found that the number of down-regulated DEGs was higher than the number of up-regulated DEGs at the 3rd and 6th MPTP/p injections, especially at 6th injection. The number of up-regulated DEGs was higher than the numbers of down-regulated DEGs at 10th MPTP/p injection (Fig. 4c). The co-occurrence DEGs in different groups was shown in Fig. 4c. All differentially expressed genes at indicated time points were listed in Tables S1-S3, and volcano plots were shown in Fig. S4.

### Three Transcriptional Response Phases in MPTP/p-Induced Progressive PD Model

**Fig. 5:**
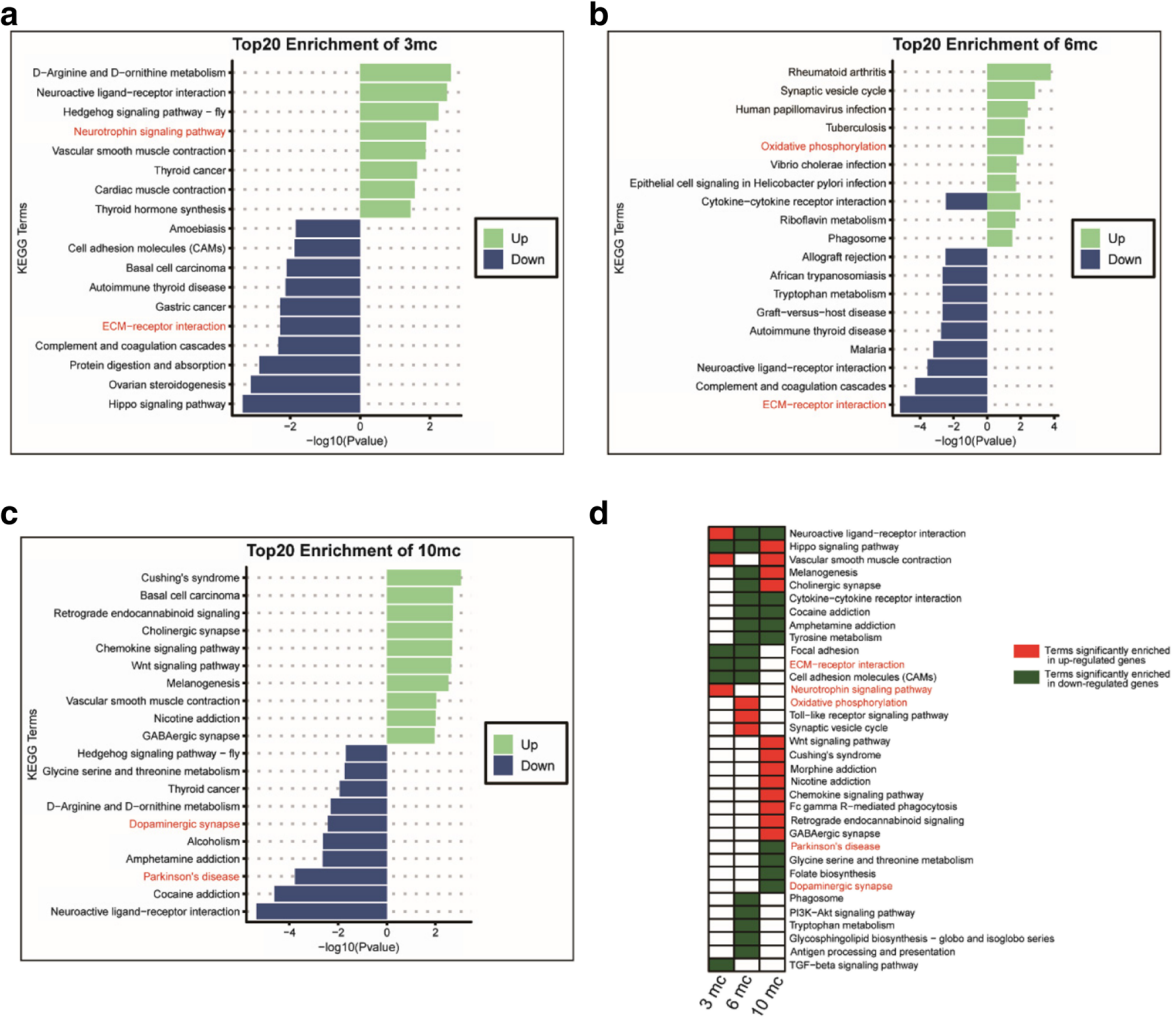
KEGG enrichment of differentially expressed genes. **a** Top 20 KEGG pathway enrichment of DEGs between the MPTP/p-induced progressive PD model group and the control group after the 3rd injection (*P* < 0.05). **b** Top 20 KEGG pathway enrichment of DEGs between the MPTP/p-induced progressive PD model group and the control group after the 6th injection (*P* < 0.05). **c** Top 20 KEGG pathway enrichment of DEGs between the MPTP/p-induced progressive PD model group and the control group after the 10th injection (*P* < 0.05). **d** Summarization of enriched KEGG terms of up or down-regulated genes among 3rd, 6th, and 10th MPTP/p injections. “mc” indicated the comparison of model and control groups. Red shading blocks indicated terms enriched in up-regulated genes; Green shading blocks indicated terms enriched in down-regulated genes

We next investigated biological process inferred by functional enrichment analysis during the injections and compared it with the corresponding saline-treatment group. In 3rd injection, up-regulated genes were enriched in terms of “neuroactive ligand-receptor interaction,” “vascular smooth muscle contraction,” especially the “neurotrophic signaling pathway” (such as *Ntf3*, *Trp73*, *Prkcd*, *odf3b*). Down-regulated genes were enriched in terms of “Hippo signaling pathway,” “focal adhesion,” “TGF-beta signaling pathway,” especially “cell adhesion molecules” and “ECM-receptor interaction pathway” (such as *Col6a2*, *Fn1*, *Sv2c*) (Fig. 5a). In the 6th injection, genes involved the “ECM-receptor interaction pathway” were also down-regulated, while genes involved “oxidative phosphorylation” were up-regulated (such as *Atp6ap1l*, *Atp6v1e1*, and *Ndufa7*) (Fig. 5b). In the 10th injection, down-regulated genes were significantly enriched in terms of “Parkinson’s disease” and “Dopaminergic synapse” (such as *Th*, *Drd2*, *Slc6a3*, and *Slc18a2*) (Fig. 5c). Up-regulated genes were enriched in terms of “Hippo signaling pathway,” “melanogenesis,” “cholinergic synapse,” “Wnt signaling pathway,” “nicotine addiction,” “chemokine signaling pathway,” “morphine addiction,” and “retrgrade endocannabinoid signaling” (Fig. 5d). We selected 16 KEGG pathways such as neurotrophic signaling pathway, ECM-receptor interaction, neuroactive ligand-receptor interaction, cell adhesion molecules (CAMs), synaptic vesicle cycle, tyrosine metabolism, dopaminergic synapse, and Parkinson’s disease; the DEGs between the MPTP/p-induced progressive PD model group and the control group relative on these pathways to make the KEGG pathway term and gene networks to display the dynamic changed at various time points. The dynamic change on relative genes to some of KEGG pathway is shown in the KEGG term-genes network in Figs. S1–S3.

### Gene Set Enrichment Analysis of PD-Related Genes

**Fig. 6:**
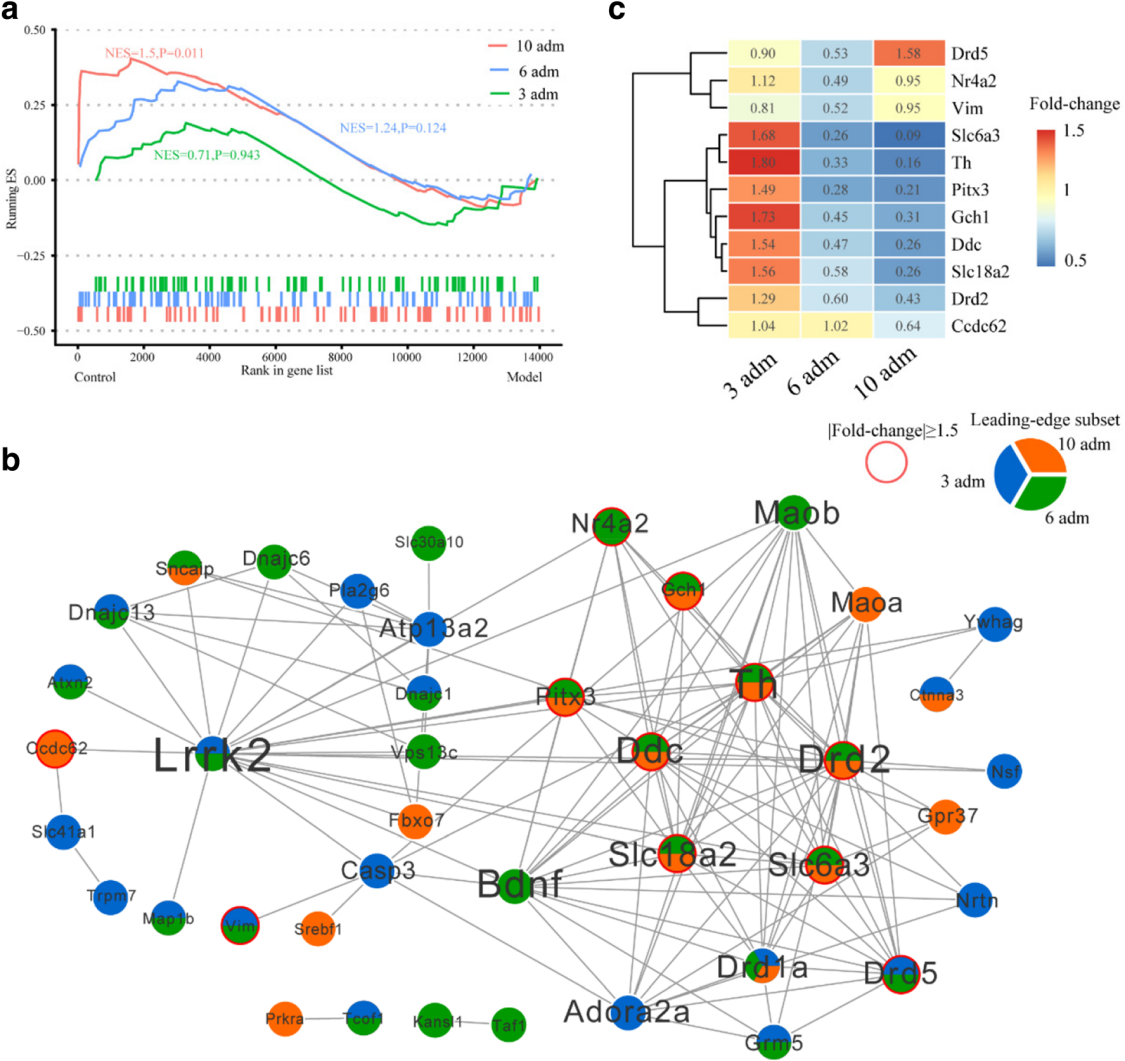
GSEA analysis of PD genes. **a** Running enrichment score (ES) in PD genes under the 3rd, 6th, and 10th injections, respectively. **b** Significant leading-edge subset genes and protein–protein interaction. Protein–protein interactions were retrieved from STRING database. Genes with fold-change ≥ 1.5 or ≤ 0.67 were shown in the red circle in either the 3rd, 6th, or 10th injection. Pie charts of node depict leading-edge subset gene present in the 3rd, 6th, or 10th injection. **c** Genes with fold-change ≥ 1.5 or fold-change ≤ 0.67 by compared model to corresponding control group. Number in cells was fold-change

Gene Set Enrichment Analysis (GSEA) is widely used for linking prior knowledge and helping uncover the collective behavior of genes in states of health and disease [28]. Here, we used GSEA to explore the relevant correlation among “Parkinson’s disease” gene set and MPTP/p injections and identify leading-edge subsets that accounts for the enrichment signal. We retrieved 100 associated genes in the term of “Parkinson’s disease” from STRING disease query using Cytoscape software with default search parameters. We termed these genes as “PD genes.” Of these, 79 human orthologous PD genes in mice were expressed in our data. “Control > Model” analyses of 3rd, 6th, and 10th injection group showed that PD genes were significantly enriched at the top of the list of 10th injection (*P* < 0.05, Fig. 6a); namely, PD genes were significantly down-regulated in 10th MPTP/p injections. We identified the leading-edge subsets consist of 22, 26, and 16 genes in 3rd, 6th, and 10th injections, respectively (Fig. 6b). *Drd1* was shared by three leading-edge subsets. Eight genes were shared by 6th and 10th injections and seven of which showed down-regulation in model group (fold-change ≥ 1.5 or ≤ 0.67; Fig. 6 b and c). Of which, five genes are related to dopamine synthesis (*Th* and *Ddc*) and transport (*Slc6a3*, *Slc18a2*, and *Drd2*).

### RT-qPCR Validation

**Fig. 7:**
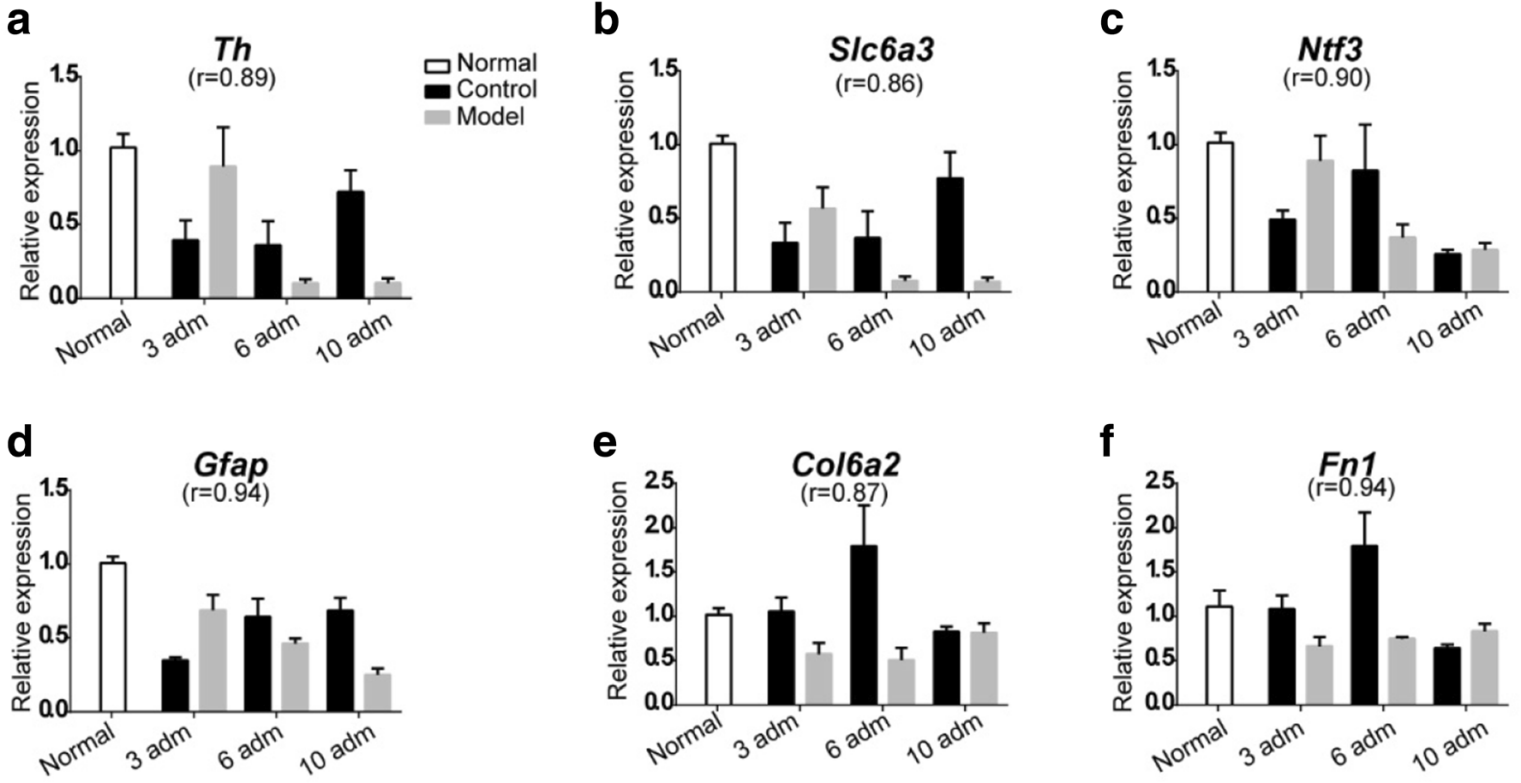
RT-qPCR validation of RNA-seq for the differential expression of six genes (**a**–**f**). “*r*” indicated the correlation coefficient between RNA-seq and RT-qPCR data

We employed RT-PCR to validate data quality. Six genes, including *Th*, *Slc6a3*, *Ntf3*, *Col6a2*, *Fn1*, and *Gfap*, were chosen for validation because these genes were associated with PD, neurotropic signaling pathway, and extracellular matrix interaction (Fig. 7). Pearson correlation coefficient (*r*) calculation showed high accordance in gene expression between RT-qPCR and RNA-seq (0.86–0.94; Fig. 7), indicating that our data was sufficient to uncover the potential molecular changes during progressive PD model.

## Discussion

PD is a neurodegenerative disease that seriously affects the quality of life. With the trend of global aging, the incidence of PD has significantly increased. At present, there is no effective disease-modifying treatment that would stop or slow down the progress of diseases. PD will remain the focus of research in the field of neuroscience for decades to come. The pathological mechanism of PD is not clear until now. Existing studies have shown that PD may be closely related to oxidative stress, glutamate receptor abnormality, ubiquitin-protease dysfunction, inflammation and cytokine activation, neurotrophic factor dysfunction, mitochondrial damage, cytoskeleton abnormality, synaptic dysfunction, and apoptotic pathway activation [13–17]. In this study, we found the dynamic changes of genes associated with pathways of the neurotropic signaling pathway, ECM-receptor interaction, oxidative phosphorylation, apoptosis, necroptosis, dopaminergic synapse, Parkinson’s disease, and other KEGG pathways, during the MPTP/p-induced progressive PD mice.

**Fig. 8:**
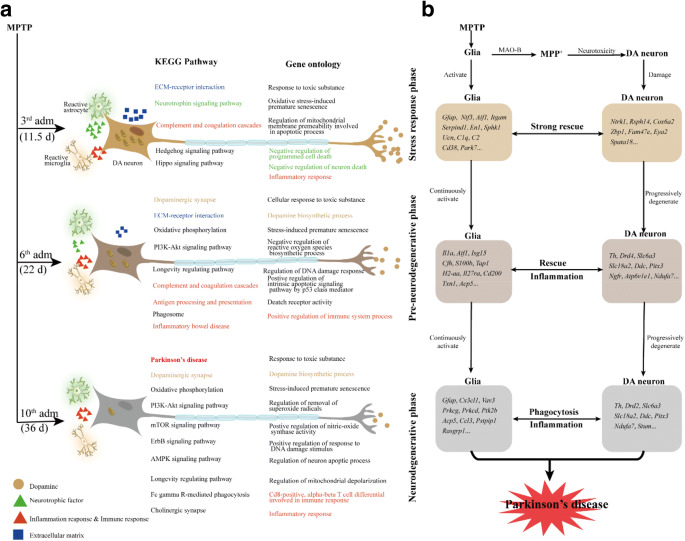
Schematic diagram illustrating the global view of transcriptional changes in MPTP/p-induced progressive PD mice model. **a** KEGG pathway and gene ontology enrichment analysis of differential expression genes during MPTP/p injections revealed three phases: stress response, pre-neurodegenerative and neurodegenerative phases. **b** Important genes are possibly related to the changes in glia cells and DA neurons in each phase

Reliable animal models are the basis of PD disease research. Many models were employed in PD research, such as toxin-induced models (especially MPTP/MPP^+^) and a-synuclein, *LRRK2*, *Parkin*, *DJ-1*, and *PINK1* genetic models [14]. Genetic animal models show low robust in cell death, at least in vertebrates, while MPTP/p-induced PD models are widely employed in the field of PD research because of similar pathological changes with PD. Further, MPTP has high lipophilicity and can rapidly cross the blood-brain barrier to enter brain. Although MPTP is not neurotoxic, it is metabolized by monoamine oxidase B in astrocytes, and terminal converted to the active toxic cation MPP^+^ (Fig. 8b). Then MPP^+^ is taken up by dopaminergic neurons through the dopamine transporter, which induces neurotoxicity primarily by inhibiting complex I of the mitochondrial electron transport chain, leading to ATP depletion and increased oxidative stress [30]. Several MPTP intoxication regimens or administration methods have been used to produce dopaminergic neurodegeneration in mice. The completion of the chronic MPTP/p progressive PD model lasted for 5 weeks in mouse, closer to the slow onset of PD in human beings. Thus, we employed this typical model to explore the neurodegenerative process of PD.

The MPTP/p-induced PD mice showed dopaminergic neurons losing in substantia nigra, lacking typical clinical symptoms consistent with PD patients [31, 32], such as stiffness, tremor, and gait. Our results were also consistent with this argument on the whole. However, we also observed some transient symptoms, tremor-like epilepsy, salivation, increased muscle tone, and backward gait in mice after several minutes of MPTP/p injection. This is similar to some clinical symptoms of PD patients, such as autonomic nervous disorders, drooling, and dystonia, but basically disappeared after a few hours (Video 1). For the behavioral tests, we found a significant increase in latency after the 10th MPTP/p injection in the olfactory experiment, and the significantly increased number of error steps during the MPTP/p injections (3rd, 6th, and 10th) in the beam transversal test. Our results indicated that PD mice exhibited significant non-motor and locomotive dysfunction after a total of 10 injections of MPTP/p. TH immunofluorescence result showed that there was no significant reduction of *TH*-positive dopaminergic neurons compared with the control group after the 3rd MPTP/p injection. However, the *TH*-positive cells in the substantia nigra were reduced to 61.9% of normal mice after the 6th MPTP/p injection, and then decreased to 51.3% after the 10th MPTP/p injection. Overall, as the MPTP/p injections increased, the number of TH-positive dopaminergic neurons decreased gradually, and the number of dopaminergic neurons was minimized after the 10th MPTP/p injection. Both the behavioral experiments and immunofluorescence results supported that the MPTP/p-induced chronic PD mouse model could achieve a steady PD-like state after the 10th injections.


ESM 6(MP4 58185 kb)

Uncovering the molecular changes in MPTP/p-induced chronic PD model will deepen our understanding of progressive processes in PD and provide potential targets for the clinical treatment on PD. KEGG pathway enrichment of DEGs between MPTP/p injections and corresponding control group showed three phases: the stress response phase, the pre-neurodegenerative phase, and the neurodegenerative phase. In the stress response phase, gene ontology analysis showed the rapid response in the tissue, including response to toxic substance, oxidative stress-induced premature senescence, regulation of wound healing, negative regulation of neuron death, and inflammatory response (Fig. 8a). We found that genes involved in the neurotrophic signaling pathway (*Ntf3*, *Prkcd*, *and Ntrk1*) were up-regulated, while genes related with the extracellular matrix-receptor interaction (*Col6a2* and *Fn1*), the apoptosis (*Lrrc74b*, *Lrrc18*, *Lmntd1*, and *Gm626*), and the necroptosis (*Hist2h2aa2*, *Zbp1*, and *Fam47e*) were down-regulated in the stress response phase. We proposed that dopaminergic neurons at this stage may be through neurotrophic, immune regulation, and other strategies to deal with exposure to MPTP/p as much as possible to maintain the microenvironment homeostasis. Next, it entered the pre-neurodegenerative phase; gene ontology analysis showed inchoate features of neurodegeneration in the tissue, including dopamine biosynthetic process, cellular response to toxic substance, regulation of DNA damage response, positive regulation of intrinsic apoptotic signaling pathway by p53 class mediator, and positive regulation of immune system process (Fig. 8a). In this phase, the expression of genes involved in extracellular matrix-receptor interaction (*Sv2c*, *Col6a2*, and *Fn1*) was down-regulated, while the expression of genes involved in the oxidative phosphorylation (*Atp6ap1l*, *Atp6v1e1*, and *Ndufa7*) was up-regulated. The expression of genes related to apoptosis (*Lrrc74b*, *Lrrc18*, *Lmntd1*, *Gm626*, and *Dthd1*) and the necroptosis (*Hist1h2ap* and *Zbp1*) was also down-regulated. At this stage, down-regulated genes accounted for the most proportion of DEGs. We speculated that at this stage, dopaminergic neurons may be struggled to the MPTP/p cytotoxicity, exhausted to achieve self-rescue, depleted of repair ability, and degenerated gradually. In the neurodegenerative phase, gene ontology showed obvious signs of PD, including dopamine biosynthetic process, response to toxic substance, stress-induced premature senescence, positive regulation of nitric-oxide synthase activity, regulation of mitochondrial depolarization, regulation of reactive oxygen species metabolic process, positive regulation of response to DNA damage stimulus, and inflammatory response (Fig. 8a). We found that PD-related genes (*Th*, *Drd2*, *Ddc*, *Pitx3*, *Slc18a2*, *Slc6a3*, *Sncaip*, *Ccdc62*, and *Fbxo7*) constituted a leading-edge subset and showed strong downregulation in the 10th MPTP/p injection when the PD model was successful achievement. In summary, dopaminergic neurons in MPTP/p-induced progressive mice may undergo degeneration and apoptosis after a series of stress, compensation, and self-rescue.

We also found that genes involved in neurotropic signaling pathway were up-regulated at the stress response phase (Fig. 8a), then down-regulated afterward. An investigation of gene expression in relatively spared parts of the substantia nigra of four PD patients indicated a reduction in neurotrophic support alternations in axon guidance cues, leading to dopaminergic cell death [16], which was consistent with our findings. Neurotrophin-3 (*Ntf3*), a neurotrophic factor, showed increased expression after the 3rd MPTP/p injections. *Ntf3* could restore synaptic plasticity in the striatum of a mouse model of Huntington’s disease [33]. *Ntf3* could activate two membrane receptors: the low-affinity receptor *p75* and the high-affinity receptor “tropomyosin receptor kinase” (*Trk*). Unlike other neurotrophic factors, *Ntf3* could activate three Trks (*Ntrk1*, *Ntrk2* and *Ntrk3*). Our data showed *Ntrk1* also increased after the 3rd MPTP/p injection. It has been shown that overexpression of *Nrtk1* in peripheral blood MSCs on PD rat model could increase dopaminergic neuron repair in lesion site [34]. Therefore, we hypothesized that *Ntf3* may bind *Ntrk1* to activate the MAPK signaling pathway, promoting cell proliferation and cell survival at the stress response phase.

Our data also showed that ECM–receptor interaction–related genes were significantly down-regulated after the 3rd and 6th injections of MPTP/p compared with the corresponding control group, including *Sv2c*, *Col6a2*, and *Fn1*. ECM molecules in the central nervous system form highly organized ECM structures around cell soma, axon initial segments, and synapses. They play prominent roles in guiding cell migration, neurite outgrowth, and synaptogenesis and regulating closure of the critical period of development, synaptic plasticity and stability, cognitive flexibility, and axonal regeneration in adults [35]. *Sv2c* modulates dopamine release and can be disrupted in Parkinson’s disease [36]. Lack of *Col6a2* leads to spontaneous apoptosis and defective autophagy in neural cells [37]. A previous study suggested that mice hippocampal neurons could elevate *Col6a2* level to activate Akt/PI3K anti-apoptotic signaling pathway for protecting against neuronal apoptosis under stress condition [38]. Another study demonstrated a neuroprotective role for *Col6a2* against the toxicity of amyloid-β peptides in Alzheimer’s disease [39]. We proposed that extracellular matrices, especially *Col6a2* and *Sv2c*, may be involved in the progression of PD.

Parkinson’s disease and dopaminergic synapse pathways were significantly enriched after the 10th MPTP/p injection (Fig. 8a). Significantly down-regulated PD genes included *Th*, *Drd2*, *Ddc*, *Pitx3*, *Slc18a2*, *Slc6a3*, *Sncaip*, *Ccdc62*, and *Fbxo7*, five of which are related with dopamine synthesis (*Th*, *Ddc*) and transport (*Slc6a3*, *Slc18a2* and *Drd2*). Dopamine (DA) plays a vital role in reward and movement regulation in the brain. In SN, DAergic neurons produce DA through tyrosine hydroxylase (*Th*) and aromatic amino acid decarboxylase (*Ddc*) or uptake DA through the transporter (*Slc6a3*) from extracellular. The newly synthesized or taken up DA is stored in vesicles with the aid of vesicular monoaminergic transporter-2 (*Slc18a2*) [40]. *Pitx3* has been implicated in the proper development of mild brain DA neurons in the substantia nigra pars compacta, and it may directly activate transcription of *Slc18a2* and *Slc6a3* [41]. Synphilin-1, a cytoplasmic protein encoded by *Sncaip*, interacts with α-synuclein, the main constituents of Lewy bodies, and plays an important role in the pathology of PD. It has been shown that Synphilin-1 has neuroprotective effects on MPP-induced PD model cells by inhibiting ROS production and apoptosis [42], suggesting that *Sncaip* may play a central role in PD. Conditional deletion of *Fbxo7* in the midbrain dopamine neurons results in an early reduction in striatal dopamine levels, together with a slow, progressive loss of midbrain dopamine neurons and onset of locomotor defects, via RPL23–MDM2–TP53 pathway [43]. Parkinson disease risk alleles in *Mapt* (rs2942168) and *Ccdc62* (rs12817488) loci were associated with global parkinsonism [44]. A research on transcriptional profile in mice with MPTP-induced early stages of PD suggested that *Drd2* may participate in the development of the compensatory mechanisms in the early stages of PD pathogenesis [45]. Also, our data-supported *Drd2* might participate in the PD progress, but on the later stages of PD.

Biological processes of transcriptional changes in MPTP/p-induced progressive PD mouse model from this study are largely consistent with the related studies on the SN of PD patients [46–48] such as dopamine metabolism, mitochondrial function, oxidative stress, and neuroinflammation, vesicular transport, and synaptic transmission. Predominant dysregulation of dopamine metabolism pathways here was also reported in several studies [49–51], specifically related genes, such as *Slc6a3*, *Slc18a2*, and *Ddc*. It should be noted that some differences are existing in molecular-level between MPTP/p-induced PD model and PD patients. Comparison of function enrichment categories of DEGs identified in multiple gene expression datasets from PD patients and MPTP mice model showed that neuronal and synaptic functions in the SN of PD patients, while cell growth and death were predominant in the MPTP mouse model [47].

Although the fact that MPTP/p-induced PD mice model could not completely replicate the features of PD, this most commonly used model provides a potential molecular basis regarding the death of dopaminergic neurons. In addition to the limitation of the model, some shortcomings still present in this study: fewer biological replicates (*n* = 3) may underestimate the DEG numbers identified in the progressive degeneration; bulk-tissue RNA-seq recovers information of the average gene expression ignoring the cell complexity and heterogeneity which hampers the identification of molecular changes generated by the dopaminergic neurons. The advanced single-cell RNA-seq technology provides the opportunity to generate transcriptomic cell profiling at cellular level, which will enable us to further study more detailed molecular changes of dopaminergic neuron loss in PD in the future.

## Conclusion

In summary, we first systematically studied the molecular toxicology process in the progressive PD model and revealed the regular patterns and network relationships between the molecular regulatory mechanisms and the biological phenotypes of the model. Our RNA-seq data revealed elevated level of neurotrophic support related genes in early stages which might partly explain little reduction of DA neurons. The dynamic change of genes was involved in pathways including neurotropic signaling pathway, ECM-receptor interaction, oxidative phosphorylation, apoptosis, necroptosis, and dopaminergic synapse. Moreover, for the first time, we proposed that transcriptional changes might reflect three phases: stress response phase, pre-neurodegenerative phase, and neurodegenerative phase, which finally led to the loss of dopaminergic neurons in MPTP/p-induced progressive PD mice. However, whether these changes were well analog with human PD and the underlying mechanism requires further in-depth investigation. This study provided a more extensive dataset of molecular toxicology for understanding the PD progress and more comprehensive theoretical knowledge for possible new treatment in the future.

## Electronic Supplementary Material

ESM 1 (XLSX 29 kb)

ESM 2 (XLSX 46 kb)

ESM 3 (XLSX 25 kb)

ESM 4 (DOCX 1471 kb)

ESM 5 (DOCX 136 kb)

ESM 7 (MP4 100255 kb)

ESM 8 (MP4 1192 kb)

## Data Availability

The datasets used or analyzed during the current study are available from the corresponding author on reasonable request. RNA-seq data were deposited in China National Genebank DataBase (CNGBdb, https://db.cngb.org/) under Project ID accession number of CNP0001036.
