# Supplementary material for: Molecular Regulatory Mechanism and Toxicology of Neurodegenerative Processes in MPTP/Probenecid-Induced Progressive Parkinson’s Disease Mice Model Revealed by Transcriptome
Source: Mol Neurobiol. 2020 Sep 30;58(2):603–16. doi: 10.1007/s12035-020-02128-5 (PMC7843579; doi:10.1007/s12035-020-02128-5)
Supplement: Supplementary file 5 — (DOCX 1471 kb) [file 12035_2020_2128_MOESM4_ESM.docx]

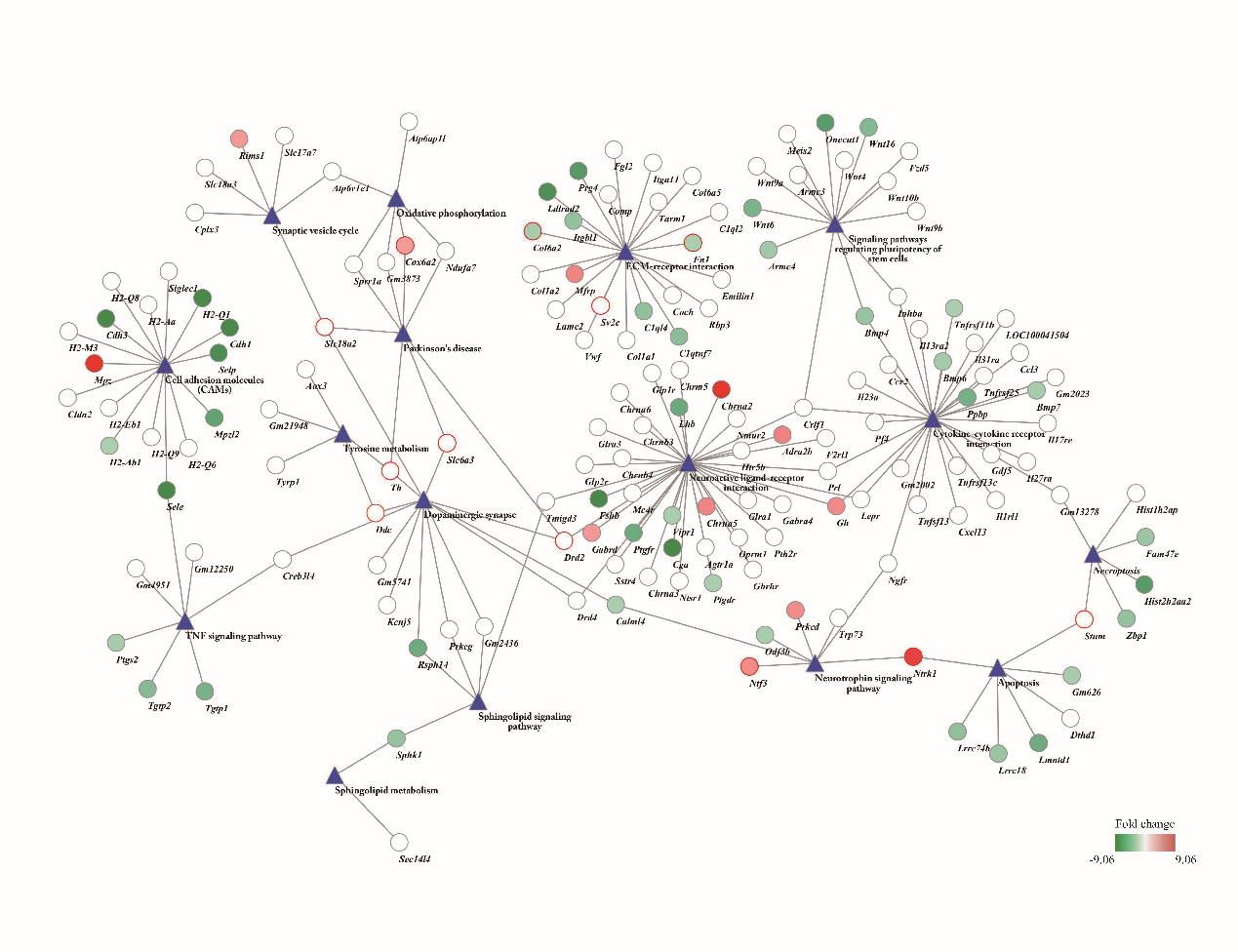


Figure S1 the KEGG term-genes network at 3^rd^ MPTP/p injections.


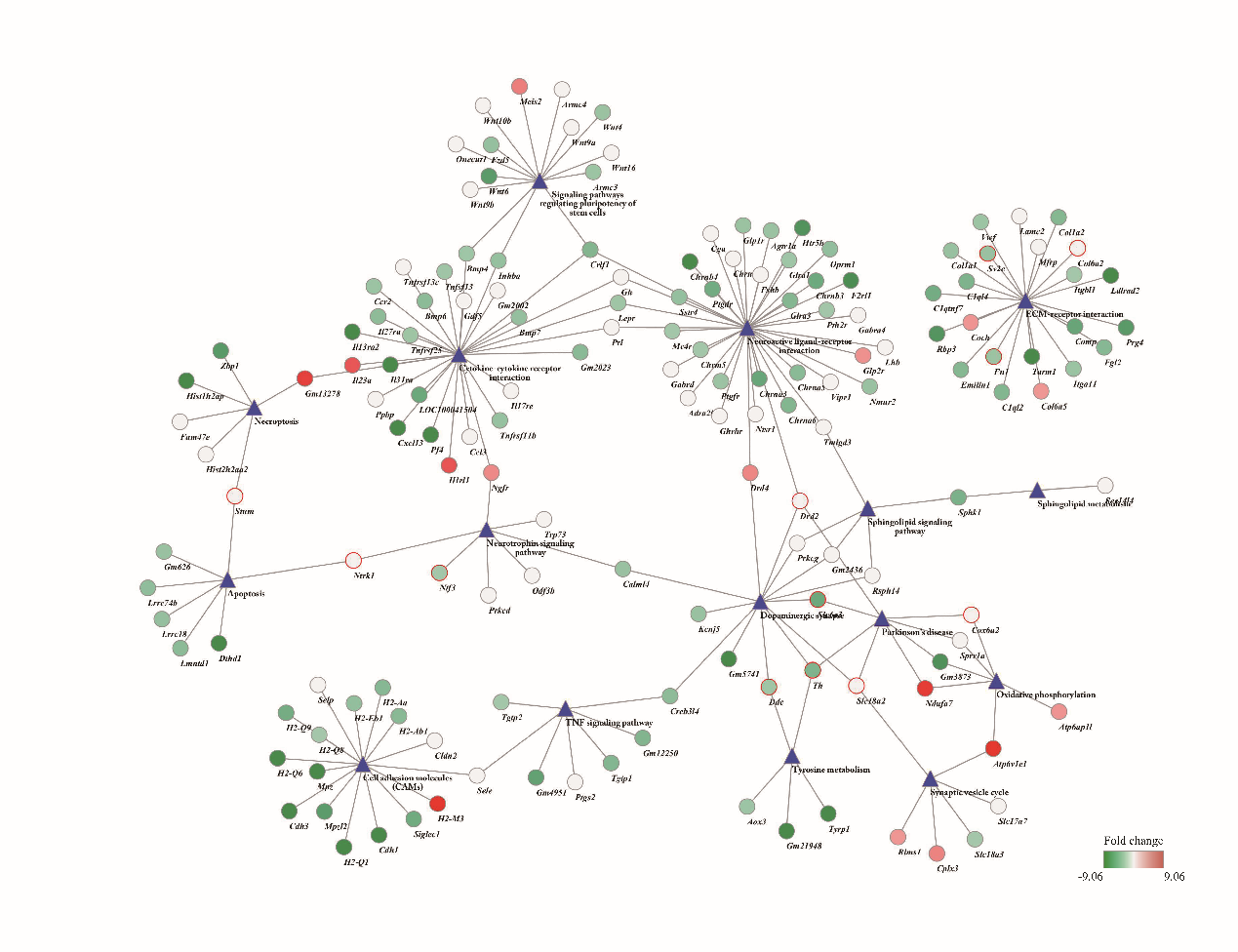


Figure S1 the KEGG term-genes network at 6^th^ MPTP/p injections.


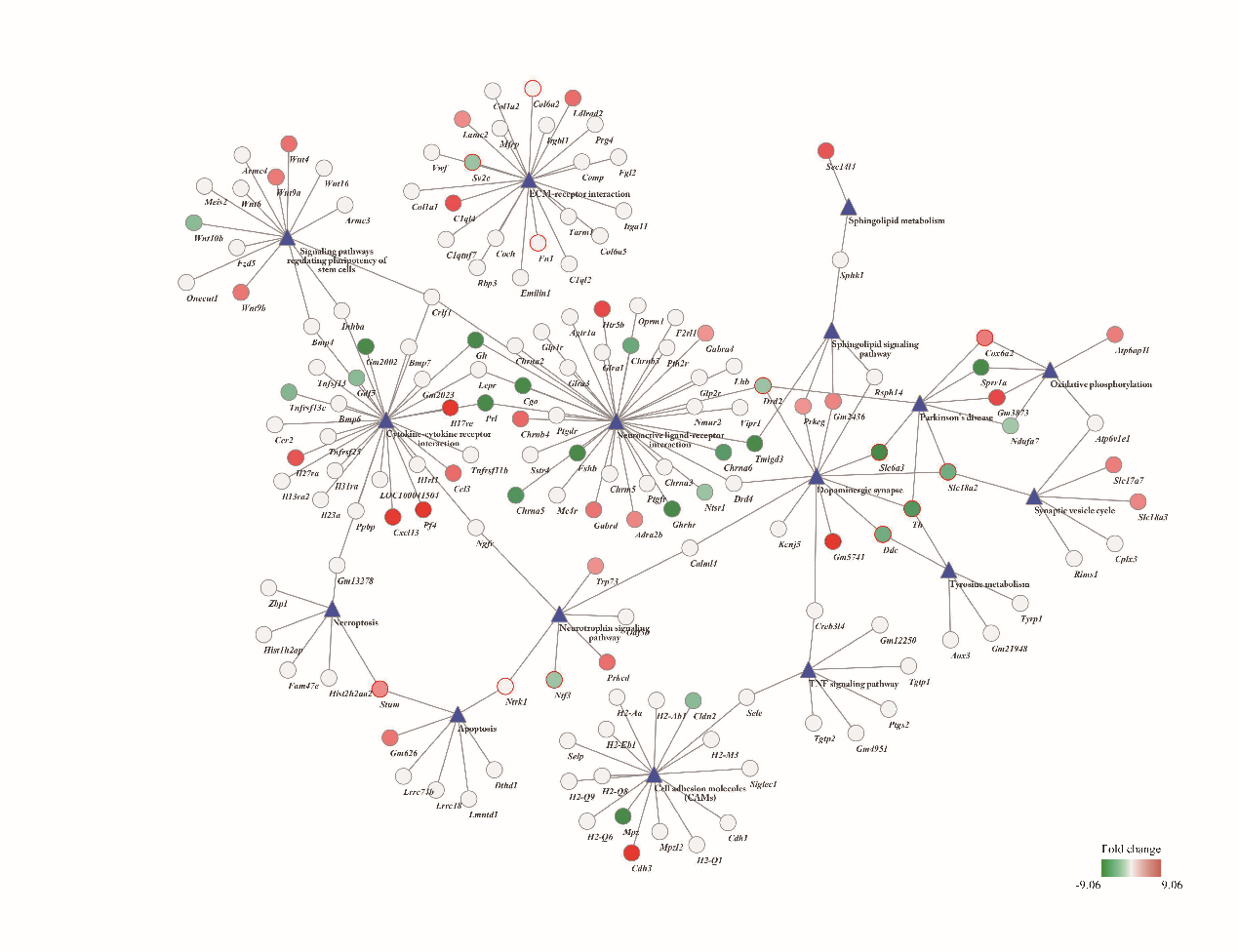


Figure S3 The KEGG term-genes network at 10^th^ MPTP/p injections.
