# Supplementary figures and images for: Molecular Regulatory Mechanism and Toxicology of Neurodegenerative Processes in MPTP/Probenecid-Induced Progressive Parkinson’s Disease Mice Model Revealed by Transcriptome
Source: Mol Neurobiol. 2020 Sep 30;58(2):603–16. doi: 10.1007/s12035-020-02128-5 (PMC7843579; doi:10.1007/s12035-020-02128-5)

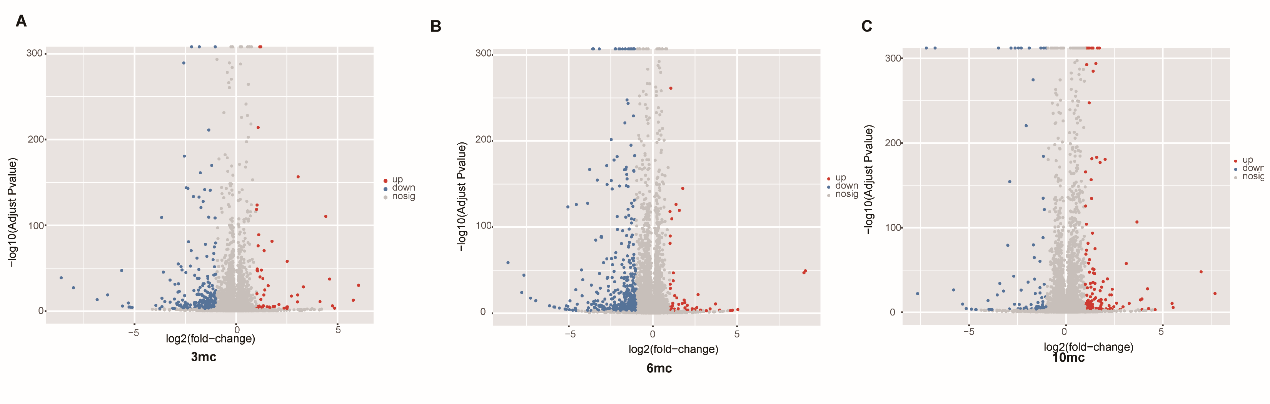


Fig. S4 volcano plots of DEGs

Supplement: Supplementary file 6 — (DOCX 136 kb) [file 12035_2020_2128_MOESM5_ESM.docx]
